# Electronic Cigarette and Atherosclerosis: A Comprehensive Literature Review of Latest Evidences

**DOI:** 10.1155/2022/4136811

**Published:** 2022-08-31

**Authors:** Vito Anggarino Damay, Setiawan, Ronny Lesmana, M. Rizki Akbar, Antonia Anna Lukito, Vita M. Tarawan, Januar W. Martha, J. Nugroho

**Affiliations:** ^1^Department of Cardiovascular Medicine, Pelita Harapan University, Banten, Indonesia; ^2^Department of Biomedical Sciences, Padjadjaran University, Bandung, Indonesia; ^3^Department of Cardiology and Vascular, Padjadjaran University, Bandung, Indonesia; ^4^Department of Cardiology and Vascular, Airlangga University, Surabaya, Indonesia

## Abstract

Coronary artery diseases (CAD), also known as coronary heart disease (CHD), are the world's leading cause of death. The basis of coronary artery disease is the narrowing of the heart coronary artery lumen due to atherosclerosis. The use of electronic cigarettes has increased significantly over the years. However, harmful effects of electronic cigarettes are still not firm. The aim of this article is to review the impact of electronic cigarette and its role in the pathogenesis of atherosclerosis from recent studies. The results showed that several chemical compounds, such as nicotine, propylene glycol, particulate matters, heavy metals, and flavorings, in electronic cigarette induce atherosclerosis with each molecular mechanism that lead to atherosclerosis progression by formation of ROS, endothelial dysfunction, and inflammation. Further research is still needed to determine the exact mechanism and provide more clinical evidence.

## 1. Introduction

Coronary artery diseases (CAD), also known as coronary heart disease (CHD), are the world's leading cause of death [1]. The basis of coronary artery disease is the narrowing of the heart coronary artery lumen due to atherosclerosis. CAD may appear as one of several manifestations, ranging from stable angina, unstable angina, myocardial infarction, and sudden cardiac death [2]. The American Heart Association reported that in 2016, 15.5 million people above 20 years old in the United States were affected by CAD, and the prevalence was shown to increase with age [3]. In Indonesia, it is suspected that by the year 2013, there will be more than 2.6 million CAD patients, and the number of cases is expected to rise significantly in recent years [4].

CAD is multifactorial, affected by both genetic and lifestyle factors [5]. One of the most significant modifiable risk factors of CAD is cigarette smoking. Cigarette smoking is a habit widely known to be harmful to health. Smokers can be differentiated into active and passive smokers depending on whether the smokers are voluntarily and directly involved with tobacco use. In recent years, many different alternatives to tobacco smoking can be found in the market, including smokeless tobacco, hookah (also known as shisha), and electronic cigarettes (also known as vaping) [6, 7]. Electronic cigarettes (e-cigarette) are especially recognized since they were marketed as a “healthier” substitute to tobacco smoking [8]. Around 2.3-3.7% million US adults were estimated to be current e-cigarette users [7, 9]. In Indonesia, 10.9% of adults were aware of the rising use of the e-cigarette, and 2.5% of them were current e-cigarette users [10]. The number of e-cigarette users increased over the years in different parts of the world, even among the youths [11, 12]. The increasing prevalence of e-cigarette users is a concerning issue since its use is related to devastating health and socioeconomic burdens. It is estimated that the general cost of e-cigarette smoking is twice higher than traditional tobacco smoking [13]. Principally, an e-cigarette is an electronic device capable of burning specialized liquids contained in a cartridge, generating aerosol and vapor smoke [14].

Smoking has been widely known to cause various complications affecting different system organs. The growing scientific evidence also implied the same findings for e-cigarette smoking. The use of e-cigarettes is recently more recognized as a cause of different public health problems. E-cigarette use causes cough and wheezing and promotes oxidative stress while generating harmful substances and wastes harmful to the environment and surrounding people [15]. E-cigarette smokers were found to inhale toxic and carcinogenic compounds in the e-cigarette vapor [16]. Overlapping with tobacco cigarette in some aspects, e-cigarettes were recorded to negatively impact respiratory, digestive, hematologic, and obstetric organ tissues while also holding the potential to cause trauma, poisoning, and allergic reaction [17]. Particularly, CAD is among the main contributors of smoking-related mortality and morbidity, and past studies found that smoking was closely associated with different aspects of CAD pathogenesis, including the formation of atherosclerosis and coronary artery spasm [18].

Considering the overlapping harmful effects of tobacco and e-cigarette smoking, we conduct this literature review study to assimilate the findings of past studies regarding the effect of e-cigarette smoking on atherosclerosis as the central pathologic basis of CAD. In this study, the term “e-cigarette smoking” encompasses both active and passive e-cigarette smokers unless elaborated otherwise. First, we will concisely describe our methodology to find works of literature that we cite in the writing of our review study. Then, we will try to outline the general cardiovascular pathologies associated with e-cigarette use and revisit the pathogenesis of atherosclerosis. After that, we will review the available literature regarding the mechanism by which e-cigarette smoking affects the development of atherosclerosis and preexisting cardiovascular diseases.

## 2. Molecular Mechanism of Atherosclerosis as the Basis of Cardiovascular Disease

Atherosclerosis is the principal pathologic factor of many different types of cardiovascular diseases [1]. The endothelial cell lining the inner surface of the blood vessels is vital in secreting chemical molecules such as nitric oxide (NO) and angiotensin II to maintain circulatory homeostasis. NO is capable of preventing vascular thrombosis and inflammation. Changes in NO formation may alter vascular physiology, and it has been noted as one of the earliest features in several vascular diseases, such as atherosclerosis. Alteration in hemodynamics may incite endothelial dysfunction, subsequently progressing into cardiovascular diseases. Several risk factors, including smoking, high cholesterol plasma levels, and obesity, may aggravate hemodynamic instability. Circulatory hemodynamics and atherosclerosis were thought to be related, and this hypothesis is proved by looking at the predilection of the atherosclerotic lesion location, which is mainly concentrated in regions with high turbulence, such as the aorta branch point. Kruppel-like factor 2 (KLF2), a protective transcription factor against atherosclerosis, is expressed when the blood flows laminarly. On the other hand, turbulent blood flow will induce the endothelial cells to activate Nf-kB inflammatory pathway and undergo subsequent cytoskeletal restructuring [19–21].

Hyperlipidemia is a prominent determinant of atherosclerosis. There are two types of lipoproteins, such as low-density lipoproteins (LDLs) that are found in the circulation, and it has been long known as highly atherogenic, and high-density lipoproteins (HDLs) are the counterpart of LDL, known to be atheroprotective. HDL is deemed atheroprotective due to its capability to carry cholesterols from peripheral circulations back to the liver to be degraded. Statin, a drug class commonly prescribed to treat hypercholesterolemia, lowers LDL levels by inhibiting HMG-CoA reductase and increasing HDL levels when combined with lifestyle changes. LDL contains single apolipoprotein B (apoB) and easy to be oxidized. Atherosclerosis progression is initiated when lipids that contain lipoproteins accumulated in the tunica intima and activated the endothelium. This process leads to inflammation that known with increase of leukocyte infiltration and growth factor production [22]. Oxidized LDL is less vulnerable to degradation, and the accumulation of oxidized LDL inside a macrophage will cause the macrophage to turn into foam cells [23]. Endothelial dysfunction is an early stage of atherosclerosis progression and risk factor of other cardiovascular diseases, such as dyslipidemia.

**Figure 1:**
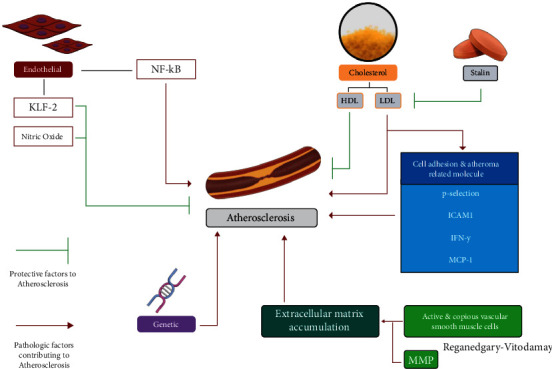
Several factors that affect atherosclerosis.

Endothelial dysfunction will also cause the secretion of inflammatory proteins. Several cell adhesion molecules (CAMs), including p-selectin and ICAM-1, will be upregulated by the inflammatory molecules. The increased cell adhesion molecules will aid leukocyte adherence and migration into subendothelial tissue. Several highly expressed molecules in the atheroma are interferon-*γ* (IFN-*γ*) and monocyte chemoattractant protein-1 (MCP-1). The secreted IFN-*γ* accelerates the progression of atherosclerosis by enhancing the formation of foam cells, while MCP-1 activates leukocyte integrin, a protein that aids cellular attachment to the endothelial surface. Macrophages that enter the intima layer of the vessel will uptake oxidized LDL and turn into foam cells while also promoting the inflammatory reaction. Macrophages aggravate the inflammation inside the atheroma by activating T cells and secreting MCP-1 and IL-1, and both molecules will trigger further upregulation of endothelial cell adhesion molecules [24]. Recently, studies have showed that endothelial dysfunction-induced smoking has a higher levels of soluble intracellular adhesion (ICAM-1), P-selectin, and E-selectin in smokers than in nonsmokers [25]. Formation of foam cells caused by the differentiation of monocyte into macrophages also promoted the chemokine secretion, such as MCP-1 and CXCL-1/2/3, leading to plaque and thrombosis formation. On the other hand, endothelial dysfunction also stimulated the proinflammatory cytokine secretion, such as IL-1Beta, IL-6, IL-8, and TNF, and activated several signaling pathways that include PI3K, Akt, and MAPK that could activate the Nf-kB as a transcription factors inducing inflammation and extracellular matrix secretion as a mediator of macrophage attachment (Figure 1) [26]. Furthermore, endothelial dysfunction also stimulated the growth factors like TGF-*β*, Fibroblast Growth Factor (FGF), and Platelet-Derived Growth Factor (PDGF) that activated Nf-kB and Smad2/3/4 signaling pathway leading to vascular smooth muscle cell proliferation and migration [27].

Vascular smooth muscle cells (VSMC) were also involved in atherosclerosis. Inactive VSMC is contractile, expressing smooth muscle myosin heavy chain (MYH11) and smooth muscle actin alpha-2 (ACTA2) [21]. Under normal conditions, VSMC can only be scarcely found inside the tunica intima of the vessels. However, the amount of VSMC inside the intima layer of atherosclerotic vessels is copious and in an active state. The VSMCs will proliferate quite rapidly and produce extracellular matrix (ECM) in that state. The accumulation of the VSMC and the ECM will increase the size of the atheroma [21, 24]. Intima and media thickening in atherosclerosis progression is an evidence that overproliferation of VSMC induced Fibroblast Growth Factor-2 (FGF-2) and enhanced the cell migration in inflammation. There are several other agents of VSMC migration both of growth factors and extracellular matrix components, such as angiotensin II, VEGF, TNF-*α*, and collagen I/IV/VIII [28].

Extracellular matrix (ECM), such as collagens and noncollagenous proteins, has been shown to influence the function and activity of vascular cells, particularly macrophages and vascular smooth muscle cells (VSCM) [29]. Cells inside the atheroma can modify surrounding ECM by utilizing matrix metalloproteinase (MMP) enzymes. MMP is a group of zinc-dependent endopeptidases capable of disintegrating ECM proteins, and macrophages are among the biggest producers of MMP enzymes. Several types of MMPs were found to accelerate the progression of atherosclerosis by unknown mechanisms. However, MMP enhanced macrophage accumulation inside the atherosclerotic plaque by releasing cytokines and growth factors inside the matrix. Furthermore, due to its capability to break down ECM, MMP was thought to play a role in atheroma destabilization [30, 31]. Atherosclerosis plaque formation and destabilization is controlled by MMP-9 activity and could be a predictor of cardiovascular mortality in patients with coronary artery disease [32].

Genetic is one of the least apparent risk factors of atherosclerosis. Some genetic-related conditions may foster atherosclerosis plaque progression, such as familial-type of hypercholesterolemia. Patients with this condition have abnormally high levels of LDL, related to the proprotein convertase subtilisin/kexin type 9 (PCSK9) and aberrations of apolipoprotein B (apoB). Mutation involving PCSK9 will hasten LDLR disintegration into LDL. And, due to the interaction between LDL receptor and apoB, mutations causing loss of function of any of both factors will reduce the degradation of LDL, increasing plasma levels of LDL [33].

### 2.1. Chemical Compound of E-Cigarettes and Its Effect on Atherosclerosis

Many studies have recently tried to shed light on how electronic cigarettes may impact different parts of atherosclerosis pathogenesis. Several chemicals in the e-cigarette that may contribute to atherosclerosis progression are nicotine, volatile organic compounds, oxidants, particulate matter, toxic gas compounds, and heavy metals. Different from the conventional cigarette, e-cigarette heats liquid containing propylene glycol, glycerin, nicotine, and flavoring molecules to form smoke that will be inhaled. Other chemicals generated in e-cigarettes are acrolein, phenols, formaldehyde, crotonaldehyde, pyruvaldehyde, acetone, and acetaldehyde [34]. The chemical compounds will vary depending on the brands and the used cartridge and refill solutions. Heavy metals may be found in trace amounts [35]. Even though the number of chemicals found in e-cigarette liquid is comparably lesser than those found in a tobacco cigarette, those chemicals may undergo chemical transformations due to heat utilized by the e-cigarette, which may be potentially dangerous [36].

### 2.2. Nicotine

The nicotine in the e-cigarette is significantly associated with several different cardiovascular outcomes, such as atherosclerosis, myocardial infarction, and stroke. Plasma nicotine levels of tobacco cigarette smokers and e-cigarette smokers were equal, raising questions about the safety aspects of e-cigarettes claimed by the manufacturers [37]. An estimated 0.5-15.4 mg of nicotine are generated in the e-cigarette vapor for every 300 puffs [38]. The chronic exposure to e-cigarettes containing nicotine leads to atherosclerotic plaque formation, proved by a study in rats that found no atherosclerosis plaque formed on rats exposed to the e-cigarette with 0% nicotine [39]. The mechanism by which nicotine induces atherosclerosis appears to be caused by the FFA release from adipocytes, disrupting endothelial integrity [40].

**Figure 2:**
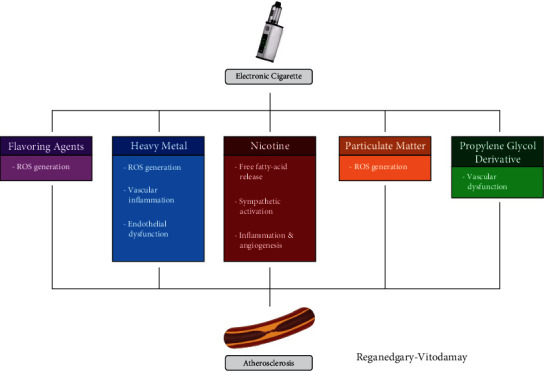
Chemical constituents of e-cigarette and their effects on atherosclerosis.

The result of past studies indicates that nicotine is a potent sympathomimetic agent capable of stimulating the sympathetic nervous system, leading to both acute and chronic shifts in blood pressure and heart rate variability [41, 42]. Nicotine may bind with nicotinic receptors found in autonomic ganglia, brain, and sympathetic nerve terminals to stimulate the release of norepinephrine and increase adrenergic activity [43]. The increasing sympathetic nerve activity may promote proinflammatory effects via the splenocardiac axis pathway (discussed below) [44]. Nicotine may activate the sympathetic nervous system via *β*_3_ receptors, inducing a proinflammatory state and ultimately increasing the proliferation of monocytes and speeding up atherosclerosis [45, 46]. Molecular mechanism of nicotine-induced atherosclerosis is when nicotine enters the endothelial cell and increase production of ROS which activates NLRP3 inflammasome that leads to the activation of caspase-1. Activation of caspase-1 will trigger pore formation of membrane, DNA fragmentation, and release of IL-1*β* and IL-18 well known as inflammation response and promoting atherosclerosis (Figure 2) [47].

### 2.3. Flavoring

Flavoring agents can usually be found in many different e-cigarette liquid brands, comprising up to 4% of the e-cigarette fluids [48, 49]. Acetoin is the flavoring agent giving rise to the “buttery” taste of e-cigarettes, while the sweet taste is usually due to diacetyl in an exceedingly high concentration [50, 51]. Several flavoring agents, such as acetylpyridine, vanillin, diacetyl, and menthol, were capable of inducing endothelial cell dysfunction in cell culture and act as oxidants and triggered the generation of ROS, ultimately leading to oxidative stress [52]. Flavorant such as acetoin and maltol were found to trigger IL-8 release by causing ROS release, shifting the immunity into a proinflammatory state [53, 54]. Previous study has shown that flavoring compounds such as menthol and eugenol induce acute alterations in endothelial cell culture and loss of nitric oxide signaling that leads to atherosclerosis formation [55].

### 2.4. Heavy Metal Particle

The vapor of e-cigarette liquid contains variations of heavy metal particles, including aluminum, cadmium, iron, nickel, and zinc, which are contaminated even further with trace metal particles from the batteries released to the vapor through the interaction with the high temperature generated during vaping [56, 57]. The most concerning heavy metal particles are those with a diameter smaller than <2.5 *μ*m, often called fine heavy metal particles, capable of accumulating in the respiratory airway and translocating into the underlying vascular tissue and causing oxidative stress formation, vascular inflammation, and endothelial dysfunction [58, 59]. High temperature in electronic cigarette well known as release of heavy metals (cadmium, chromium, and lead) from the coil into the e-liquid and exposure to these metals can cause inflammation [60]. Recently, studies have shown that exposure of cadmium induces vascular dysfunction in the aorta of mice and increases the risk of atherosclerosis [61].

### 2.5. Particulate Matters

Particulate matters are an air-suspended liquid and solid phase particle with different chemical characteristics. In general, particulate matter can be differentiated into primary and secondary particulate matter depending on the source of the particles [62]. Particulate matters are hypothesized to affect cardiovascular directly by increasing reactive oxygen species (ROS) generation, and excessive ROS levels were linked to altered vascular functions, inducing the death of vascular cells and disrupting arterial vasoconstriction and vasodilation [63]. Based on aerodynamic diameters, particulate matters (PMs) are classified into 4 types: thoracic particles (PM > 10 *μ*m), coarse particles (PM2.5–10 *μ*m), fine particles (PM < 2.5 *μ*m), and ultrafine particles (PM < 0.1 *μ*m). Animal studies investigated about particulate matter (PM2.5) on atherosclerosis and resulting elevation of LDL, MDA, and TNF-Alpha levels and reduction of superoxide dismutase (SOD) [64].

### 2.6. Propylene Glycol

Propylene glycol is the main constituent of most e-cigarette liquids, comprising 95% liquid formulation [65, 66]. When exposed to heat, propylene glycol may disintegrate into secondary products, including formaldehyde, acetaldehyde, and methylglyoxal [67]. Methylglyoxal was found to disrupt vascular functions and accelerate atherosclerosis [68]. Acrolein is another oxidant found in the e-cigarette liquid and has been widely studied due to its carcinogenicity [35]. Chronic exposure to acrolein was correlated with cardiovascular events [69]. Formation of thermal dehydration products from propylene glycol highly depends on temperature. High temperature will produce more aldehyde products and increase the cardiovascular disease risk [70]. Exposure of electronic cigarette that contains 70% of propylene glycol (PG) 1 hour/day and 6 days/week for 4 weeks resulted in cardiac fibrosis [71]. Furthermore, 50% propylene glycol exposed in mice induces vascular oxidative stress [72].

### 2.7. Association of Electronic Cigarette and Atherosclerosis

**Figure 3:**
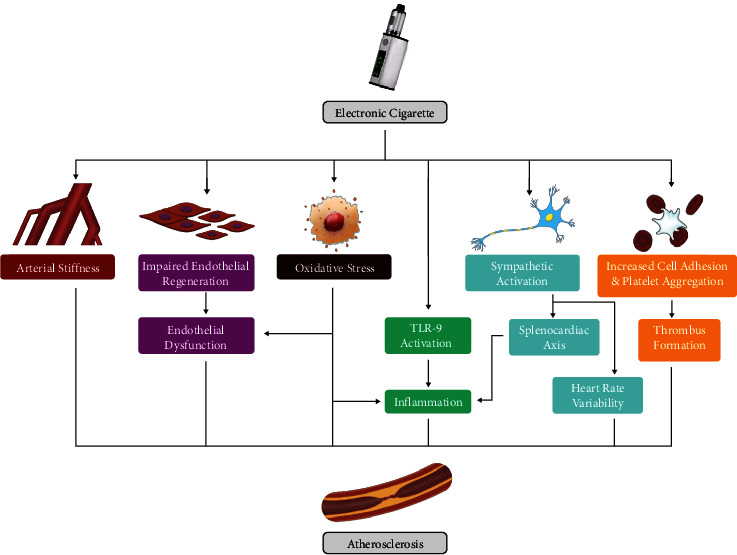
Mechanisms by which e-cigarette contributes to the pathogenesis of atherosclerosis.

Arterial stiffness is associated with alterations of stress features due to modification of structural components. This modification involves a complex interaction, such as cell signaling pathways that lead to alteration of collagen and glycoproteins of the extracellular matrix of the artery wall [73]. E-cigarettes and their constituents contribute to the pathogenesis of atherosclerosis by causing different types of pathologic features of atherosclerosis. Arterial stiffness is one of the biomedical indicators of arterial wall inflammation (Figure 3). It may be measured using a PET scan to detect the increasing metabolic activity of the inflamed arterial wall segments [74]. The findings of some studies indicated that the use of e-cigarettes is associated with increased stiffness of aortic and other arterial vessel walls [42, 74–76]. Decreased arterial stiffness has been associated with an increased risk of cardiac events [77].

Endothelial dysfunction has been accepted as the most crucial aspect also known as early stage in the atherosclerosis progression and the ensuing cardiovascular diseases [78]. Microscopically, the characteristic of endothelial dysfunction is the disrupted regeneration of the endothelial cells and the endothelial-dependent vasodilatory mechanism [79]. In the context of scientific research, endothelial dysfunction may be approximately evaluated by measuring flow-mediated dilatation (FMD) of the brachial artery. Acute use of e-cigarettes resulted in similar FMD impairment compared to the acute use of tobacco cigarettes [75, 80]. The endothelial dysfunction induced by e-cigarette smoking is comparable to tobacco cigarette smoking, and both are capable of significantly inducing endothelial inflammation and dysfunction [80–82]. Endothelial dysfunction may also be indirectly measured by evaluating circulating endothelial progenitor cell (EPC). Previous study was found that ten puffs from an e-cigarette are enough to increase the amount of EPC in circulation [41].

Tobacco smoking is known to increase oxidative stress, and so does e-cigarette smoking [83]. Recent literature discovered that e-cigarette aerosol generates oxidative stress comparable to tobacco smoke, and the reactive oxygen species (ROS) and oxidants generated by both cigarette types are similar [84]. ROS are a group of chemicals with unpaired electrons on its outermost shell, thus damaging biochemical compounds found on the surface and inside the cells. Billions of free radicals are contained within each puff of e-cigarette, and exposure to e-cigarette vapor will trigger ROS release inside the body [85, 86]. Breach of cellular antioxidant defense due to ROS may cause DNA damage [87]. The oxidization damage was thought to be caused by nicotine to a certain extent; on the other hand, the use of e-cigarettes containing nicotine was found to acutely increase plasma levels of myeloperoxidase, a type of oxidative stress marker [75].

Oxidative stress has been widely known to be closely related to endothelial dysfunction. It also has been closely associated with inflammation, another major contributor of atherosclerosis, via the peroxidation reactions, activating the monocytes and ultimately causing vessel wall inflammation [88]. Atherosclerosis has been accepted as an inflammatory disease for some period. Exposure to e-cigarette vapor is linked to inflammation and leukocyte activation [89, 90]. The concept of the splenocardiac axis has been suggested as the central inflammatory mechanism in the pathogenesis of atherosclerosis. This concept stands on the evidence of the connection between the sympathetic nervous system, spleen, and bone marrow as a cause of atheroma formation [44]. The increased sympathetic nerve activity will activate progenitor cells residing in the bone marrow due to acute stress introduced, and the progenitor cells will migrate into the spleen and undergo maturation into proinflammatory monocytes [45, 91]. The monocytes will then enter the circulatory system and work together with oxidants and other prothrombotic factors to speed up atherosclerosis. These pathological changes of the proinflammatory state induced by e-cigarette use persisted for several weeks to months [44, 92]. The physiological basis of the splenocardiac axis was confirmed using ^18^F-FDG-PET/CT in several studies, a noninvasive radiologic modality used to detect active inflammation [93]. It was observed that there is an increased plasma CRP level and higher inflammatory process inside the spleen after an attack of the acute coronary syndrome, which may also be an independent future cardiovascular event [44, 74, 94].

Toll-like receptors (TLRs) are an essential group of the innate immune system that recognize each pathogen-associated molecular pattern [31]. Toll-like receptor 9 (TLR9) is one of the innate immunity receptors that is expressed on macrophages. Activation of TLR9 has been implicated in inducing IL-6 and tumor necrosis factor *α* (TNF-*α*) secretion, vascular wall inflammation, and the transformation of macrophages into foam cells [95]. Furthermore, TLR9 plays a crucial role in the development of vascular inflammation through proinflammatory activation of macrophages in angiotensin II-induced atherosclerosis via MyDD8 pathway [31, 96]. Histopathologically, monocyte infiltrating the atherosclerotic lesion is one of the most significant components of atheroma [97]. Additionally, drug intervention focusing on blocking TLR9 hampers the progression of atherosclerosis progression, supporting the notation that TLR9 is associated with atherosclerosis [98].

Platelet plays a central role in forming thrombus in coronary artery disease. E-cigarette vapor was observed to induce platelet activation and aggregation. The use of e-cigarettes increased the formation of soluble CD40-ligand and P-selectin while also increasing platelet aggregation significantly in less than 5 minutes after single e-cigarette use [8]. The CD40 expressed on the platelet affects atherosclerosis by mediating the leukocyte recruitment and chemokine release, playing vital roles in atherosclerotic plaque formation [99]. The CD40 also transiently expressed on T cells under inflammatory conditions, especially in tumors signaling pathway [100]. Meanwhile, P-selectin is a cellular adhesion molecule involved in procoagulant activities and leukocyte activation.[101] One in vitro study found that harmful components found in the e-cigarette vapor significantly cause platelet activation, with a degree of effect compared to traditional tobacco cigarette smoking. The extent of platelet activation was found to be independent of nicotine concentration [102]. Another study conducted by Qasim et al. [103] noted the increasing activity of platelets exposed to in vitro e-cigarette vapor inhalation system with denser *α*-granule secretion, higher phosphatidylserine expression, and higher activation of Akt, ERK, and *α*IIb*β*3 integrin. However, these findings still need further confirmation as very few studies on the impact of e-cigarette use on platelet function.

The sympathetic nerve regulates the physiology of the cardiovascular system. Recent evidence consisted of findings regarding the hyperactivated sympathetic state in e-cigarette users, affecting several aspects of cardiovascular health such as blood pressure [104]. Heart rate variability (HRV) measures sympathetic nervous system hyperactivation, and HRV has long been associated with cardiovascular events [105]. In chronic e-cigarette users, aspects of HRV pointed to the predominant activation of the nervous system accompanied by decreased vagal tone, a pattern known to be involved in recent and future cardiovascular events [106]. The change in sympathetic nerve activity contributes to the incidence of CAD by hastening the progression of atherosclerosis via the splenocardiac axis. The increasing sympathetic nerve activity triggers an inflammatory pathway contributing to atherosclerosis via the release of proinflammatory monocytes by the spleen [75].

### 2.8. E-Cigarettes: Future Considerations

The emission of e-cigarette vapor has given rise to several environmental concerns. Even though the number of chemicals contained in e-cigarette vapor was less than tobacco cigarette smoke [107], the dangerous chemicals contained within e-cigarette smoke may cause adverse effects to people surrounding the smoker. The particulate matter in the e-cigarette smoke may contaminate the active smoker's air and cause systemic effects if inhaled by the passive smokers, especially the smaller fraction of particulate matter [108]. The propylene glycol emitted by electronic cigarettes has a shorter half-life time (10-20 seconds) than propylene glycol emitted by tobacco cigarettes (1.4 h); it still poses a dangerous threat when vaping is done in a small room with poor ventilation [66].

Information gathered by studies regarding the impact of e-cigarette vapor to passive surrounding passive smokers may be of value to determine the future direction of public health policy and regulatory developments [109]. Since the beginning of their creation, the e-cigarette has been claimed as a healthy alternative to tobacco smoking, developed to help in smoking cessation [110]. In Indonesia, a study by Aminullah forecasted that the rising use of e-cigarettes would replace traditional tobacco cigarette smoking, which will be influenced by tax incentives, technological development, and stricter tobacco regulation by the government [111]. Public exposure to e-cigarette advertisements and marketing is associated with an increased chance of e-cigarette smoking on nonsmokers, adolescents, and youth due to the illusion of “perceived safety” of e-cigarettes.[11, 112]Thus, government-imposed marketing restriction on e-cigarettes was thought to be beneficial in decreasing e-cigarette consumption. Other perceived factors contributing to the increased use of e-cigarettes are the customization options available to suit personal preference and the similarity of e-cigarettes to tobacco cigarettes compared to traditional nicotine replacement therapy [12]. However, due to its copious harmful effects, the use of e-cigarettes as a substitute for tobacco cigarette smoking is still not recommended since the harms outweigh the questionable benefits compared to the standard nicotine replacement therapy. E-cigarettes should be studied further before being widely implemented as an alternative to standard nicotine replacement therapy in routine clinical practice.

## 3. Conclusion

E-cigarette is associated with several noxious compounds, such as nicotine, propylene glycol, particulate matter, heavy metals, and flavorings, that lead to atherosclerosis progression. Every single one of these toxic compounds has its own molecular mechanism associated with cardiovascular disease risk factor by ROS formation, endothelial dysfunction, and inflammation. Even though e-cigarettes are believed to be the safer substitute for tobacco smoking, several studies proved that it is still potentially harmful. Further research is still needed to determine the clinical use and harms of e-cigarettes and their effect on cardiovascular system.
